# Superspreading of SARS-CoV-2 Omicron BA.2.23 among vaccinated Finnish adults: symptomatic COVID-19 only contracted by those without recent infection

**DOI:** 10.1017/S0950268823001024

**Published:** 2023-07-04

**Authors:** Marianna Riekkinen, Mikael Kajova, Mari Eriksson, Annika Luukkainen, Ville Holmberg, Tuomas Aro, Sari H Pakkanen, Simo Miettinen, Reetta Montonen, Teemu Smura, Tinja Lääveri, Anu Kantele

**Affiliations:** 1 Department of Infectious Diseases, Inflammation Centre, Helsinki University Hospital and University of Helsinki, Helsinki, Finland; 2 Human Microbiome Research Unit, University of Helsinki, Helsinki, Finland; 3 Meilahti Vaccine Research Centre, MeVac, University of Helsinki and Helsinki University Hospital, Helsinki, Finland; 4 Department of Virology, Faculty of Medicine, University of Helsinki, Helsinki, Finland; 5 HUS Diagnostic Centre, HUSLAB, Clinical Microbiology, University of Helsinki and Helsinki University Hospital, Helsinki, Finland; 6 Department of Computer Science, Aalto University, Espoo, Finland

**Keywords:** COVID-19, hybrid immunity, Omicron, SARS-CoV-2, superspreading event

## Abstract

An outbreak of SARS-CoV-2 was confirmed after an academic party in Helsinki, Finland, in 2022. All 70 guests were requested to fill in follow-up questionnaires; serologic analyses and whole-genome sequencing (WGS) were conducted when possible.

Of those participating – all but one with ≥3 vaccine doses – 21/53 (40%) had test-confirmed symptomatic COVID-19: 7% of those with earlier episodes and 76% of those without. Half (11/21) were febrile, but none needed hospitalisation. WGS revealed subvariant BA.2.23.

Compared to vaccination alone, our data suggest remarkable protection by hybrid immunity against symptomatic infection, particularly in instances of recent infections with homologous variants.

## Introduction

Equipped with a remarkable ability to immune evasion, the SARS-CoV-2 Omicron variant and its sublineages are transmitted more effectively than the previously emerged variants [1, 2]. Extensive transmission may take place at any large gathering – even those including well-vaccinated individuals. Numerous outbreaks have been reported after various social occasions such as a Christmas get-together in Norway [3] and a private gathering in the Faroe Islands [4].

We describe here an outbreak following a party with 70 adults in May 2022. Extensive transmission was revealed afterwards, when many of the guests had symptoms typical of COVID-19 and positive SARS-CoV-2 test results. In addition to the outbreak data, we describe the results of virus whole-genome sequencing (WGS) and variant-specific neutralising antibodies analyses for a subset of the participants.

## Methods

Held in a 59 m^2^ (approx. 160 m^3^) banquet hall, the academic party comprised dinner, multiple speeches, dancing, and singing. The first Omicron wave – BA.1 from mid-December 2021 to mid-March 2022 and BA.2 thereafter – had just receded [5], and national guidelines no longer advised the use of masks in gatherings; testing for SARS-CoV-2 was recommended only if symptoms manifested, and outbreak reporting was no longer mandatory.

### Questionnaire

Participants were requested to fill in a web survey (Webropol) covering background information, receipt of SARS-CoV-2 vaccine doses, prior COVID-19 infection episodes confirmed by RT-PCR and/or rapid diagnostic (antigen) tests (RDT), behaviour at the party (use of alcohol, socialising, dancing), ensuing COVID-19 symptoms, and test results. An attack rate was determined for contracting symptomatic COVID-19 at the party. A 9-month follow-up questionnaire collected information on vaccinations and PCR/RDT-confirmed infections.

### Serological analyses and whole-genome sequencing of RT-PCR samples

Some of the participants had provided blood samples in a Clin_COVID-19 master study [6, 7], which served as baseline for serological analyses. Post-party blood samples were requested from 36 participants who had reasonable access to a sampling facility.

The levels of anti-S1 IgG antibodies in sera were measured with an enzyme-linked immunosorbent assay (ELISA) according to Jalkanen et al. protocol [8]. The amounts of neutralising antibodies (NAbs) against Wuhan-Hu-1, Beta, Delta, and Omicron variants were analysed by a pseudovirus-neutralising assay [9] using ACE2 and TMPRSS2-expressing human embryonic kidney cells (HEK293-ACE2-TMPRSS2). Titres of <1:20 were defined as negative and titres of 20–40 as low, 80–320 as moderate, and ≥ 640 as high levels of NAbs.

If available, nasopharyngeal swab samples taken for RT-PCR analysis were subjected to whole-genome sequencing, as previously described [10].

### Ethical statement

All participants signed a written informed consent to participate in the Clin_COVID-19 master study exploring symptoms and immune responses [6, 7]. The study protocol had been approved by the HUS Ethics Committee (HUS/1238/2020). The questionnaire was slightly modified to include a few additional party-related questions.

### Statistical analyses

Pearson’s chi-square test or Fisher’s exact test was used to compare categorical variables when applicable; for continuous variables we applied Mann–Whitney-U-test. Statistical significance was defined as p < 0.05 or ORs with 95% CIs ranging either above or below 1. The analyses were carried out using SPSS 28 software (IBM Corp., Armonk, NY).

## Results

### Study population and symptoms

Of the 70 guests, 53 were included in our study: at the time of the party 21 already participated in Clin_COVID-19 [6, 7] and the remaining 32 were recruited afterwards. Here we only focus on transmissions at the party; no one wore a mask.

**Figure 1.**
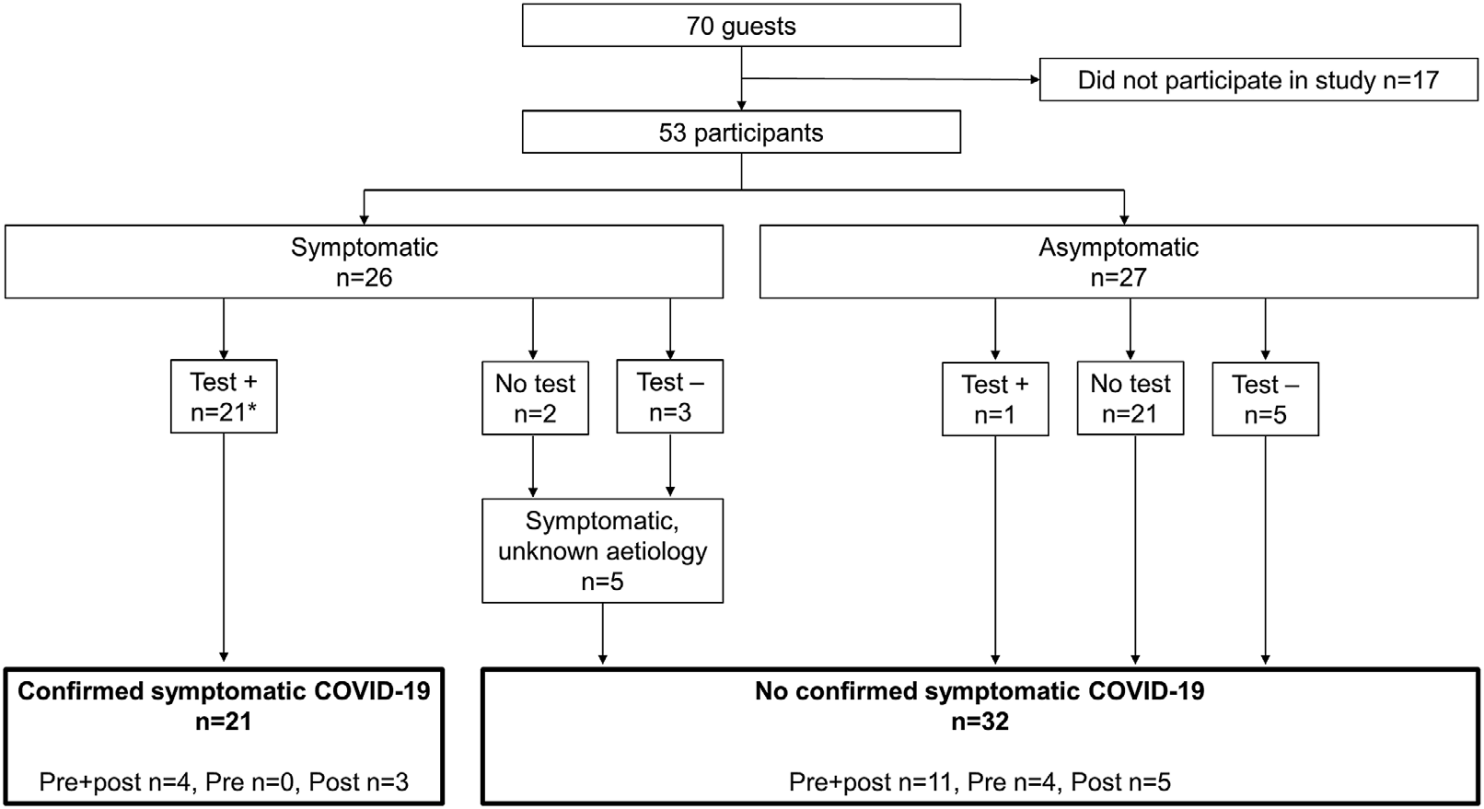
Distribution of participants in groups Confirmed symptomatic COVID-19 and No confirmed symptomatic COVID-19, and samples available for serological analyses. Test: SARS-CoV-2 RT-PCR and/or rapid detection (antigen) test (RDT). Pre + post: participants with both baseline and post-party blood samples for serological analyses. Pre: participants with only baseline blood samples for serological analyses. Post: participants with only post-party blood samples for serological analyses. Confirmed symptomatic COVID-19: participants who developed symptoms and tested positive for SARS-CoV2 on days 1–7 after the party. Symptomatic, unknown aetiology: participants who developed symptoms and were not tested or tested negative for SARS-CoV2 on days 1–7 after the party. No confirmed symptomatic COVID-19: Symptomatic participants with unknown aetiology together with all participants that remained asymptomatic on days 1–7 after the party regardless of test status. *Number of positive tests on days 1–7 after the party (Day 0) were: 9/Day 3, 8/Day 4, 3/Day 5 and 1/Day 6.

On days 1–7 after the party, 49% (26/53) reported symptoms. The 21 symptomatic participants with positive RDT or RT-PCR tests constituted the group Confirmed symptomatic COVID-19, suggesting an attack rate of 40%. Five symptomatic participants with unknown aetiology together with all asymptomatic participants were included in the group No confirmed symptomatic COVID-19 (n = 32, 60%) (Figure 1). For age and sex distributions in the groups, see Table 1. Two participants were immunocompromised and two ≥80 years of age. Symptomatic COVID-19 was reported by parties at all seven dinner tables. No associations were found between behaviour (alcohol consumption, socialising, dancing), and infection risk (data not shown).

**Table 1.** Demographics, SARS-CoV-2 vaccinations, and prior COVID-19 infection episodes of groups Confirmed symptomatic COVID-19 and No confirmed symptomatic COVID-19

	Total	Confirmed symptomatic COVID-19^a^	No confirmed symptomatic COVID-19^b^		
	n (%)	n (%)	n (%)	p-value	OR (95% CI)
**Total**	53	21 (39.6)	32 (60.4)		
**Age,** median (IQR)	53 (47–59)	60 (52–68)	50 (42–54)	0.130	
**Sex**					
Female	35 (66.0)	17 (48.6)	18 (51.4)		ref.
Male	18 (34.0)	4 (22.2)	14 (77.8)	0.063	0.3 (0.08–1.1)
**Prior Covid-19 infection**					
Yes	28 (52.8)	2 (7.1)	26 (92.9)		ref.
No	25 (47.2)	19 (76.0)	6 (24.0)	<0.001	41.2 (7.5–226.7)
**Immunity score** ^c^					
3	22 (41.5)	18 (81.8)	4 (9.1)	ref.	ref.
4–5	31 (58.5)	3 (9.7)	28 (90.3)	<0.001	42.0 (8.4–210.1)
**Time since latest Covid-19 infection** (if applicable)					
Days median (IQR)	81 (58–126)	455 (139–770)	81 (55–108)	0.085	
≤120 days	21 (75.0)	0 (0)	21 (100)		
>120 days	7 (25.0)	2 (28.6)	5 (71.4)	0.056	NA
**Time since latest vaccination**					
Days median (IQR)	225 (189–242)	222 (217–226)	225 (182–242)	0.623	
≤120 days	3 (5.7)	1 (33.3)	2 (66.7)		ref
>120 days	50 (93.4)	20 (40.0)	30 (60.0)	1.000	1.3 (0.1–15.7)
**Time since latest Covid-19 infection or vaccination**					
Days median (IQR)	81 (58–126)	183 (139–226)	81 (55–108)	<0.001	
≤120 days	24 (45.3)	1 (4.2)	23 (95.8)		ref
>120 days	29 (54.7)	20 (69.0)	9 (31.0)	<0.001	51.1 (5.9–439.3)

Abbreviations: ref. = reference category. NA = not applicable.

a Group Confirmed symptomatic COVID-19 comprises all participants who developed symptoms and tested positive for SARS-CoV2 on days 1–7 after the party. The attack rate for contracting symptomatic COVID-19 is the number of participants in this group divided by the total number of participants.

b Group No confirmed symptomatic COVID-19 comprises all asymptomatic participants regardless of SARS-CoV2 test status (negative n = 5, positive n = 1, not tested n = 21) together with symptomatic participants who were not tested (n = 2) or tested negative (n = 3) on days 1–7 after the party.

c One point for each received vaccine dose and/or prior COVID-19 infection episode.

The test-confirmed symptomatic participants reported following solicited symptoms: sore throat 62% (13/21), rhinitis 57% (12/21), cough 52% (11/21), fever 52% (11/21), fatigue 43% (9/21), myalgia/arthralgia 33% (7/21), headache 29% (6/21), excessive sleepiness 29% (6/21), weakness 24% (5/21), loss of appetite 14% (3/21), impaired concentration 14% (3/21), and impaired olfaction 10% (2/21). Vertigo, loose stools, paraesthesia, impaired stress tolerance, irritability, and melancholy were each reported by one participant. One immunocompromised participant with confirmed symptomatic COVID-19 was given intravenous remdesivir treatment in the hospital for the prevention of severe disease.

### Previous SARS-CoV-2 vaccinations and COVID-19 episodes

In early May 2022, the prevailing national guidelines recommended three vaccine doses to all adults and a fourth one to special risk groups (those strongly immunocompromised, the elderly at nursing homes, and all ≥80 year-old). Of our participants, 92% (49/53) had received three and 6% (3/53) four vaccine doses; the single one with two doses was also considered fully immunised on account of having had two COVID-19 episodes. Only three participants – all in the special risk groups – had their latest (fourth) vaccine dose administered within 4 months (24–46 days before the party), one of them reporting symptomatic test-confirmed disease. The latest type of vaccine appeared not to influence infection risk: 58% of those (14/33) given BNT162b2 and 35% (7/20) of those given mRNA-1273 had symptomatic test-confirmed COVID-19 (*p* = 0.592; OR = 2.1; 95%CI 0.2–21.3).

In total, 53% (28/53) reported prior COVID-19 infections, most episodes (83%, 25/30) having occurred during the first Omicron wave that began in December 2021 by BA.1 followed by BA.2 [5]. For the time span since our participants’ latest COVID-19 episode or vaccination, see Table 1. Symptomatic test-confirmed COVID-19 was now reported by 7% (2/28) and 76% (19/25) of those with and without prior infections, respectively. The two re-infected participants – both having received three vaccine doses – reported previous episodes 770 and 139 days earlier (latter individual immunosuppressed).

In February 2023, 51/53 participants filled in the 9-month questionnaire. One reported RDT-confirmed COVID-19 ten days after the party (presumable transmission from their spouse having contracted COVID-19 at the party). Two reported PCR-confirmed COVID-19 episodes at 5 months when BA.5 predominated [5], the other febrile but not requiring hospitalisation. Neither had contracted symptomatic disease at the party.

### Sequencing data and serological analyses

In the four RT-PCR samples available, identical genome sequences of the BA.2.23 Omicron sublineage were detected.

Baseline serum samples were available for 19 participants (for 18, 3–136 days before and for one, five days after the party), while 23/36 provided sera specimens 21–34 days after the party. Both baseline and post-party sera were available for 15 participants (Figure 1).

In the baseline, the neutralising antibody (NAb) titres against Omicron proved high (≥1:640) for 53% (10/19), moderate (1:80–1:320) for 26% (5/19), and low (1:20–1:40) for the rest. None of those with high titres had symptomatic, test-confirmed COVID-19; 90% (9/10) reported a previous infection episode in 2022 and one no earlier episodes. All four with low baseline Omicron titres had at least a moderate NAb titre against wild-type virus (1:160–1:640); two with symptomatic test-confirmed disease after the party and the other two with disease after baseline sampling but before the party. In post-party samples, high titres (≥1:640) against Omicron were seen for 96% (22/23) and a moderate (1:80) in one participant. All but one (22/23) of those with high titres reported having contracted COVID-19 either at the party or earlier.

Of the 15 individuals providing paired samples, eight had ≥2-fold titre rise in NAb against Omicron, four with symptomatic test-confirmed infection after the party and four uninfected but with a recent infection before the party.

Alongside higher titres against Omicron, increases were seen in cross-reactive NAbs to wild-type, Beta VOC, and Delta VOC. Serology results by infection status are shown in the Supplementary table.

## Discussion

Superspreaders and superspreading events are considered central to the dissemination of SARS-CoV-2 [11]. Despite presumedly high background immunity, SARS-CoV-2 subvariant BA.2.23 showed extensive propagation at the party, with an attack rate of 40% for symptomatic test-confirmed COVID-19.

Our outbreak resembles the superspreading at a Norwegian Christmas celebration soon after Omicron had emerged [3], where a cohort of highly vaccinated individuals contracted SARS-CoV-2 with an attack rate as high as 74%. Likewise, in the Faroe Islands, an Omicron outbreak with an attack rate of 63.6% was reported following a private gathering of health-care workers with three prior vaccine doses [4]. Such superspreading events among fully vaccinated individuals demonstrate the remarkable capacity of emerging variants to overcome vaccine-induced immunity elicited by a wild virus-based regimen.

In this study, a recent episode of COVID-19 – presumably caused by homologous Omicron BA.1 or BA.2 – together with earlier heterologous, wild virus-based vaccinations apparently protected against symptomatic BA.2.23 infection. Although recent homologous infection appeared to have a great impact, the underlying heterologous vaccination presumably enhanced the protection it afforded. Of our vaccinated participants, symptomatic test-confirmed disease was reported by 76% of those without and 7% with a prior COVID-19 episode (*p* < 0.001; OR 41.2; 95%CI 7.5–226.7). Apart from the risk group patients, all our participants had received their latest doses of the heterologous wild virus-based vaccine over 4 months earlier, while most of their previous infections had occurred within 4 months when Omicron BA.1 and BA.2 – homologous with BA.2.23 spreading at the party – predominated [5]. For the two confirmed cases re-infected despite three vaccine doses and a prior infection, the other was immunosuppressed and for the other infection-induced immunity was elicited by a wild-type, that is, heterologous virus 2 years earlier [5]. Both time elapsed and homology with the immunising variant probably contributed to protection.

Other studies have examined the dynamics of hybrid immunity elicited by the combination of vaccines and COVID-19 episodes. In Qatar, vaccine efficacy against symptomatic disease among participants with three BNT162b2 doses (median of latest one 43 days earlier) was 52.2% among those without and 77.3% with a prior COVID-19 episode [12]. These findings accord with WHO’s recent systematic report suggesting that hybrid immunity confers both stronger and longer-lasting protection against Omicron than vaccination alone [13]. However, protection duration depends not only on the waning of immunity – whether vaccine- or infection-induced – but also the emergence of new variants overcoming previous immunity [14, 15]. In a recent report on triple-vaccinated individuals, previous Omicron infection provided 97.1% protection against the BA.2 subvariant [15]. As pointed out by Goldberg et al. regarding natural and hybrid immunity, it is difficult to demonstrate which is more critical, homology of (sub)variant or time span since infection [14].

In our data, none of those with symptomatic test-confirmed COVID-19 (presumed BA.2.23) reported a reinfection over our 9-month follow-up when BA.5 predominated. It should be pointed out that despite their limited efficacy against transmission of current variants, wild virus-based COVID-19 vaccines confer longer-lasting protection against severe disease and mortality [13]. Consistently, none of our participants developed symptoms severe enough to require hospitalisation.

### Serology

Our serology results were logical: the disease was not contracted by those with high baseline NAb titres against Omicron and, in paired samples, a rise in NAb titres was seen after infection. Furthermore, the increase in Omicron-specific titres was also reflected as a rise against wild-type and prior VOCs.

### Limitations

The most obvious limitation of our study is the low number of participants and serologic samples, and many untested individuals. Since most asymptomatic participants were not tested, we could not evaluate the factual attack rate nor hybrid immunity against asymptomatic infection. Some symptomatic COVID-19 cases may have been missed: single negative RDTs may not have been reliable, and some symptomatic participants were not tested. Recall bias concerning the timing of previous COVID-19 episodes was deemed unlikely, since test dates of PCR-confirmed infections were available in health records, and participants were likely to have recorded the dates of RDT-confirmed infections to schedule their subsequent booster vaccinations. As obvious, asymptomatic infections and symptomatic ones contracted outside the party cannot be ruled out, yet the latter was considered improbable, since the timing of symptom onset accorded with the disease contracted at the party. In spite of these limitations, however, our major findings on symptomatic test-confirmed COVID-19 appeared evident.

### Conclusions

Despite the great efficacy of the various SARS-CoV-2 vaccines in preventing severe COVID-19, their success against transmission remains limited. Besides illustrating the remarkable transmission capacity of the Omicron BA.2 subvariant, our outbreak report suggests a considerable increase in protection against symptomatic COVID-19 afforded by hybrid immunity compared to vaccination alone – 7% versus 76% infected. This conclusion appears evident at least in instances of infection episodes having occurred over the past few months or with homologous infecting variants. It is impossible to disentangle which is more critical, homology of variant or time span since natural or vaccine-induced immunisation.

## Data Availability

We report a small outbreak with potentially recognisable participants; to protect their anonymity, individual-level data are not provided.

## Abbreviations

COVID-19: Coronavirus disease 2019
ELISA: Enzyme-linked immunosorbent assay
NAb: Neutralising antibody
RDT: Rapid diagnostic (antigen) test
RT-PCR: Reverse transcription polymerase chain reaction
SARS-CoV-2: Severe acute respiratory syndrome coronavirus 2
WGS: Whole-genome sequencing

## Supplementary material

For supplementary material accompanying this paper visit https://doi.org/10.1017/S0950268823001024. click here to view supplementary material

## Key finding

COVID-19 outbreaks can also occur among highly vaccinated individuals.

Hybrid immunity appears superior in protection against symptomatic COVID-19 infection.

Recent infections with homologous variants appear to afford particularly good protection.
