# Supplementary material for: Superspreading of SARS-CoV-2 Omicron BA.2.23 among vaccinated Finnish adults: symptomatic COVID-19 only contracted by those without recent infection
Source: Epidemiol Infect. 2023 Jul 4;151:e113. doi: 10.1017/S0950268823001024 (PMC10368952; doi:10.1017/S0950268823001024)
Supplement: Supplementary file 1 [file S0950268823001024sup001.docx]

**Supplementary table.** Serology results for party participants grouped by status of prior and party-derived COVID-19.

|  | Total  n | Days from latest COVID-19 vaccine dose, median (IQR) | Days from latest COVID-19 before party, median (IQR) | Baseline titre against wild-type virus  (n= 19) | Titre against wild-type virus  after party  (n=23) | Baseline titre against Omicron  (n= 19) | Titre against Omicron  after party  (n=23) |
| --- | --- | --- | --- | --- | --- | --- | --- |
| No COVID-19 infection before party; infected at party | 6 | 238 (234–241) |  | 1:160 –  1:320 | >1:2560 | <1:20 –  1:80 | 1:2560 –  >1:2560 |
| No COVID-19 infection before party; not infected at party | 2 | 216 (199–232) |  | 1:2560 | >1:2560 | 1:640 | 1:1280 |
| COVID-19 infection before party and before baseline serology; infected at party | 1 | 226 | 770 | >1:2560 | >1:2560 | 1:160 | 1:2560 |
| COVID-19 infection before party and before baseline serology; not infected at party | 9 | 225 (225–340) | 81 (73–105) | 1:2560 – >1:2560 | 1:1280 – >1:2560 | 1:320 –  >1:2560 | 1:80 –  >1:2560 |
| COVID-19 infection before party but after baseline serology; not infected at party | 5 | 243 (242–244) | 60 (19–74) | 1:640 –  1:1280 | >1:2560 | 1:40 –  1:640 | 1:640 –  1:2560 |
| COVID-19 infection before party, no baseline serology, not infected at party | 4 | 231 (176–244) | 66 (25–121) |  | >1:2560 |  | 1:2560 –  >1:2560 |

IQR = interquartile range
